# The Impacts of Along-Channel Acupuncture on the Protein Expressions of the Chloride Channel of the Rats with Myocardial Ischemia

**DOI:** 10.1155/2013/321067

**Published:** 2013-06-24

**Authors:** Ze-Dong Cheng, Chun-Ri Li, Xiao-Jiao Shao, Pei-Jing Rong, Xiao-Qing Zhang, Fan-Rong Liang, Yuan Li, Yi-Guo Chen

**Affiliations:** ^1^Liaoning University of Traditional Chinese Medicine, Shenyang 110032, China; ^2^Institute of Acupuncture, China Academy of Chinese Medicine, Beijing 100700, China; ^3^Chengdu University of Traditional Chinese Medicine, Chengdu 610075, China

## Abstract

Recent evidence suggests that chloride (CL^−^) channels are involved in myocardial ischemia. In this study, the impact of acupuncture on the protein expressions of Cystic Fibrosis Transmembrane Conductance Regulator (CFTR) and CLC-2 CL^−^ channel of the rats with myocardial ischemia were tested and its mechanism was explored. The rats for experiment were distributed randomly into 5 groups: blank control group, modeling control group, Neiguan (PC-6) treatment group, Lieque (LU-7) control group, and Non-acupoint control group. The rats of all groups, except the blank control group, had myocardial ischemia via multiple subcutaneous injection of isoproterenol (ISO). Electroacupuncture treatment was given to Neiguan (PC-6) treatment group, Lieque (LU-7) control group, and Non-acupoints control group, respectively, once a day for 7 days. The results show that acupuncture can alleviate the myocardial ischemia of cardiac tissue, decrease significantly the activities of serum SOD and MDA, and thereby influence the protein expressions of CFTR and CLC-2 in CL^−^ channels. The results of the study implies that acupuncture suppresses the pathological changes of cardiac tissue of rats with myocardial ischemia and regulates the protein expression of CFTR and CLC-2 CL^−^ channels, which may serve as one possible mechanism to reduce myocardial ischemia.

## 1. Introduction

Long-term myocardial ischemia is a key cause for ischemic heart diseases, and a number of studies were conducted on myocardial ischemia. There are many ion channels involved in the pathogenesis of ischemic heart diseases [1]. However, the relationship between cardiovascular disease and function of chloride channel has not been identified. Some of the recent studies explored the protective effect of acupuncture on myocardial ischemia [2]. In some studies [3], CFTR CL^−^ channels are taken as novel therapeutic targets for the treatment of ischemic cardiac diseases. Clinically Acupuncture has been applied widely to treat ischemic heart diseases for a long time, and Neiguan (PC-6) acupoint, as the classic acupuncture point, chosen to apply acupuncture in these treatments. Experimental research shows that acupuncture has cardioprotective effect on rat's acute myocardial ischemia, and acupuncture on Neiguan (PC-6) may benefit the treatment of myocardial ischemia [4]. However, the underlying mechanism remains unknown. This study is to provide the experimental evidence of the cardioprotective effect of acupuncture in treating myocardial ischemia. As shown in research, acupuncturing Neiguan (PC-6) acupuncture point can change the activities of serum SOD and MDA and the indicators of myocardial ischemia. This study explores the effect of acupuncturing Neiguan (PC-6) on myocardial ischemia treatment and its working mechanism, based on the existing theories and research findings on the effect of acupuncture on myocardial ischemia and employing immunoblotting technique to observe the protein expressions of CFTR and CLC-2 CL^−^ channels.

## 2. Materials and Methods

### 2.1. Animals

Male adult Sprague-Dawley rats, weighing 240 ± 10 g, were used in the experiment. Upon arrival, animals were given one week to adjust to the new environment (20 ± 1°C, 45–60% relative humidity, white noise db, and 12/12 h light/dark cycle with the light from 6:00 AM to 6:00 PM), with food and water available freely prior to experimental procedures. All experimental procedures were performed during the light cycle. The animal care procedures were carried out in accordance with the National Institutes of Health Guide for the Care and Use of Laboratory Animals. Every effort was made to minimize their suffering.

### 2.2. Experimental Procedure

#### 2.2.1. Models Preparation

All rats were randomly divided into the following five groups (*n* = 10 per group): (1) blank control group (normal control with no modeling intervention or treatment); (2) modeling control group (only received injection of ISO for modeling); (3) Neiguan (PC-6) treatment group (received injection of ISO for modeling and electroacupuncture at the Neiguan (PC-6) acupoint); (4) Lieque control group (received injection of ISO for modeling and electroacupuncture at the Lieque (UL-7) acupoint); and (5) nonacupoints control group (received injection of ISO for modeling and electroacupuncture at the middle of TianShu and ShenQue points). Except for those in the blank control group, rats were fixed at the supine position and anaesthetized with an intraperitoneal injection of pentobarbital sodium (35 mg/kg). Then, they were connected to the BL-420S biological and functional experimental system to record the normal ECG of rats. After a multipoint subcutaneous injection (on limb roots and back, 5-6 points in total, injection completed within 10 seconds) of ISO (85 mg/kg, two shots in total, 24 hours apart each shot), the model of rat myocardial ischemia was occupied and the II Lead Electrocardiograph (ECG) was recorded, respectively, at the first, the fifth, the tenth, and the fifteenth minute after injection. Following that, the changes of Point J (the cross of QRS wave group and wave T) in each group were observed, and the standard of a successful myocardial ischemia modeling was set when there is a visible decrease [5], as shown in Table 1.

#### 2.2.2. Electroacupuncture Treatment

After the successful modeling was established, acupuncture was applied at Neiguan (PC-6) treatment group, Lieque (UL-7) control group, and nonacupoint control group, respectively. For Neiguan (PC-6) treatment group and Lieque (UL-7) control group, the acupuncture points were chosen based on Acupuncture Points Diagram of Experimental Animals established by Association of Experimental Acupuncture Research (AEAR) of the National Association of Acupuncture (NAA). For nonacupoint group, acupuncture was applied at the point in middle of TianShu and ShenQue acupoints. 

For Neiguan (PC-6) treatment group and nonacupoint and Lieque (UL-7) control groups, the rats were fixed at supine position and connected to G6805-D electroacupuncture apparatus with *ϕ* 0.35 × 15 mm acupuncture needles once a day. The needle was inserted obliquely to form an angle of approximately 30 degrees against the skin along the meridian which the acupoint belongs to and twisted into the rat skin for 2 mm with density wave at 2–20 Hz and till the limb of rat quivered appropriately and lasts for 20 minutes each time. The modeling control group did not receive any acupuncture, but the rats were fixed in the same way as the groups above did, once a day for the duration of 20 minutes each time. 

The rats of each group were anaesthetized with 10% chloral hydrate after 7 days since acupuncture started, the blood samples of the rats in each group were collected and marked with Abdominal Aortic Blood Collection Method, and their heart tissues were taken for backup. Four rats with induced myocardial infarction died during the modeling whereas two rats in the blank control group died of anaesthesia during operation for sampling. The operative mortality was 12%.

### 2.3. Staining Rat Myocardial Tissue with HE

Myocardial tissue was cut into suitable pieces, stained with standard HE staining method, and then observed. The stained tissues were studied by light microscopy.

### 2.4. Assay of SOD Activity

SOD activity was assayed using Total Superoxide Dismutase Assay kit with WST-1 following the manufacturer's instructions. Samples (20 uL) were used for kinase activity.

### 2.5. Assay of MDA Content

MDA content was assayed using Lipid Peroxidation MDA Assay kit following the manufacturer's instructions. Samples (0.1 mL) were used for kinase activity.

### 2.6. Protein Determination

The protein expressions of Cystic Fibrosis Transmembrane Conductance Regulator (CFTR) and CLC-2 CL^−^ channel with Western Immunoblotting Technology (Western Blot) in each group were assayed. First of all, pyrolysis liquid was put into the rat myocardial tissue based on the ratio of 1 : 10 between pyrolysis liquid and the weight of rat myocardial tissue, and homogenate after its full cracking with 12000 r/min 4°C centrifugation was obtained with the supernatant as the total protein. After separated by SDS-PAGE electrophoresis, the protein extracted from the sample was transferred onto the membrane by Western Blot. After the protein expression of CFTR and CLC-2 in CL^−^ channels was assayed with CFTR and CLC-2 antibody, the contents with reference to the known protein were determined.

### 2.7. Statistical Analysis

All results were expressed with means ± standard deviation. Group differences were evaluated using one-way analysis of variance (ANOVA). The SPSS 17.0 was used for analysis, and *P* < 0.05 and *P* < 0.01 were adopted as the statistical significance levels.

## 3. Results

### 3.1. Observation and Comparison of Stained HE Rat Myocardial Tissue Samples among Each Group (Figure 1)

In the blank control group, myocardial fibers are in an ordered arrangement with morphological integrity, full nucleus, and clear boundary, which are visible under twenty-time light microscope. In the modeling control group, the Lieque (LU-7) control group, and the nonacupoint control group, cardiac tissue was observed with the assistance of HE staining. Results showed that myocardial fibers were in disordered arrangement and fracture, sarcoplasm swelling, and obvious inflammation cell infiltration even though there were many brown granules widely distributed in the nucleus. Compared with modeling control group, the changes of cardiac tissue were clearly reduced in Neiguan (PC-6) treatment group under the same physical microscope.

### 3.2. The Impact of Acupuncturing Different Acupoints on the Serum SOD Activity in Rat (See Table 2)

Compared with the blank control group, the serum SOD levels of the modeling control group, the Lieque (LU-7) control group, and the nonacupoint control group were significantly lower (*P* < 0.01). Further comparison with the modeling control group showed that the levels of serum SOD were also significantly increased in the Neiguan (PC-6) treatment group (*P* < 0.01). No differences were found between the modeling control group, the Lieque (LU-7) control group, and the nonacupoint control group (*P* > 0.05).

### 3.3. The Impact of Acupuncture at Different Acupoints on the Contents of Serum MDA in Rats (Table 3)

Compared with the blank control group, the serum MDA contents of the modeling control group, the Lieque (LU-7) control group, and the nonacupoint control group were significantly higher (*P* < 0.05). Further comparisons with the modeling control group showed that the contents of serum MDA were also significantly elevated in the Neiguan (PC-6) treatment group (*P* < 0.05). No differences were found between the modeling control group, the Lieque (LU-7) control group, and the nonacupoint control group (*P* > 0.05).

### 3.4. The Impact of Acupuncturing Different Acupoints on the Protein Expression of CFTR and CIC-2 CL^–^ Channel in the Rat Myocardial Muscle (See Figures 2 and 3)

Compared with blank control group, there was a significant increase in the contents of CFTR, CLC-2 CL^−^ channel protein in the modeling control group, the Lieque (LU-7) control group, and the nonacupoint control group (*P* < 0.01). Further comparison with the modeling control group showed that the contents of CFTR, CLC-2 CL^−^ channel protein was also significantly elevated in the Neiguan (PC-6) treatment group (*P* < 0.01). No differences were found between the modeling control group, the Lieque (LU-7) control group, and the nonacupoint control group (*P* > 0.05).

## 4. Discussion

According to classic theories of Traditional Chinese Medicine (TCM), acupuncturing Neiguan (PC-6) acupoint may be applied for treating acute myocardial ischemia, based on its connection with the chest discomfort and cardiodynia. As illustrated in *Experience on Acupuncture and Moxibustion Therapy*, acupuncture at Neiguan (PC-6) can be adopted to treat the sudden pain in the heart, fear, upset, and dementia. In* Compendium of Acupuncture and Moxibustion*, it is also maintained that acupuncture at Neiguan (PC-6), Daling (PC-7), or Quze (PC-3) may serve the purpose of treating pain in the chest and heart. It is also used to treat many kinds of heart diseases in clinical treatment. A number of clinical studies indicate a better curative effect on the treatment of acute myocardial ischemia that can be reached by acupuncturing at Neiguan (PC-6), as it has a tranquilizing effect and can relieve clinical symptoms of breast-pang patients and improve ischemic ECG [6]. It is confirmed in animal experiments that electroacupuncture at Neiguan (PC-6) acupoint can improve the function of left ventricular, myocardial microcirculation, and oxygen supply, besides reducing and narrowing the degree of myocardial ischemia. For acute ischemic heart disease, electroacupuncture at Neiguan (PC-6) acupoint can improve hemodynamic disturbance, increase myocardial tension, adjust the contractility, and correct arrhythmia [7]. 

Modeling rats with acute myocardial injury caused by isoproterenol (ISO) were used in this study since the model is one of those classical method to study the effect of antimyocardial ischemia [8]. Injecting a large dose of ISO continuously may lead to a myocardial ischemic injury as ISO is a *β* receptor excitomotory. The present study confirms the changes of ECG, myocardial metabolism, and histopathology of the rats injured by a large dose injection of ISO, similar to a natural occurrence of myocardial ischemic injury [9]. 

Superoxide dismutase (SOD) is an important antioxidant enzyme in cardiac muscle, which removes the surplus oxygen free radicals (OFR), protects other antioxidant enzymes from ORF inactivation, and reduces the damage. The activity of SOD indirectly reflects the ability of the body to remove OFR with a negative correlation [10]. The experiment result shows that the content of rats serum SOD is clearly increased in the Neiguan (PC-6) treatment group (*P* < 0.01), but there was no significant difference in the Lieque (LU-7) control group and nonacupoint control group compared with the modeling control group (*P* > 0.05). This indicates that acupuncture at Neiguan (PC-6) can effectively control the degree of rat myocardial damage and significantly increase the serum SOD content and the activity of SOD and thereby reduce the damage of oxygen free radicals to myocardial cells. While no such effects can be achieved by acupuncturing at Lieque acupoint or nonacupoint points.

MDA is the end product of oxygen free radical and lipid peroxidation and reflects the extent of myocardial injury. The results of this study show that the content of serum MDA in rats is clearly decreased in the modeling control group, the Lieque (LU-7) control group, and the nonacupoint control group (*P* < 0.05), compared with the blank control group. Compared with the modeling control group, the content of serum MDA in rats in the Neiguan (PC-6) treatment group is significantly reduced (*P* < 0.05), but no clear changes were observed in the Lieque (LU-7) and nonacupoint control groups (*P* > 0.05). 

There are many kinds of ion channels in myocardial cell, which play an important role when there are hypoxia, swelling, or endogenous catecholamines releasing caused by regional myocardial ischemia in heart. It is proved in recent studies [11, 12] that the chloride channels are involved in the protection against myocardial ischemia and reperfusion. The first cardiac CFTR CL^−^ channel of cDNA gene encoding cloned Riordan discovered in the year 1989 is one of phosphorylation dependent epithelial cells chloride channel [13]. The loss or dysfunction of CFTR channel may cause a variety of diseases, as shown in research [14]. CLC-2 inwardly rectifies chloride current obtained from the atrial and ventricular muscle records of mice and guinea pigs, as proved by Duan et al. in 1999 [15]. It plays an important role in the electrical activities of heart the same as cationic inward rectifier channels. The study shows that the protein expression of CFTR and CLC-2 CL^−^ channel in the modeling control group and Lieque (LU-7) and nonacupoints control groups is clearly higher than that in Neiguan (PC-6) treatment group and blank control group (*P* < 0.05), but there is no difference between the modeling, Lieque, (LU-7) and nonacupoints control groups (*P* > 0.05). The results of the current study show that the myocardial ischemia of rats may be caused by an increased concentration of protein CFTR and CLC-2 after activating chloride channel of myocardial tissue. Blocking of CL^−^ channel by acupuncture at Neiguan (PC-6) can reduce the concentration of protein CFTR and CLC-2, which could be one mechanism to treat rat myocardial ischemia. 

Acupuncture points are the specific sites where the qi of zang-fu organs and channels is transported to the surface of body. Acupuncture points are not only the reflecting places of disorders but also the sites to receive the stimulation by acupuncture and moxibustion for treatment. In history, Chinese medical scientists have observed and theorized the significant role that channels play in connecting the acupoints on body surface and the internal organs, which has been widely applied in clinical practice for nearly two thousand years.

Neiguan (PC-6) acupuncture point is a Luo-connecting point of Pericardium Meridian of Hand Jueyin. It is not only one of the eight confluence points but also a key point in Yinwei Meridian, which stops the pain and soothes heart and chest; thus acupuncture at Neiguan (PC-6) can treat the pain of heart and chest caused by blood stasis. And Pericardium Meridian, known as the protector of heart, can regulate the function of heart. As shown in the results of this study, acupuncture at nonacupoint or Lieque (LU-7) cannot treat rat myocardial ischemia as effectively as acupuncture at Neiguan (PC-6) can do. As one of the Hand Jueyin Pericardium Meridian points, acupuncturing Neiguan (PC-6) has a visible effect in treating ischemia via regulating the protein expression of chloride channel in myocardial tissue.

In summary, acupuncture at Neiguan (PC6) on rats with myocardial ischemia reflects the treatment efficacy of acupuncturing along channels. One of its treatment mechanisms can be interpreted by its adjustment of the protein expression of chloride channels CFTR and CLC-2 in myocardial cells, as proved in this study.

## Figures and Tables

**Figure 1:**
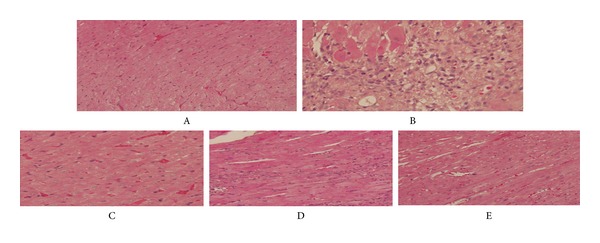
Staining results of HE in rats myocardial tissue. (A) control group, (B) modeling group, (C) Neiguan (PC-6) group, (D) Lieque (LU-7) group, (E) nonacupoint group; the same in Figures 2 and 3.

**Figure 2:**
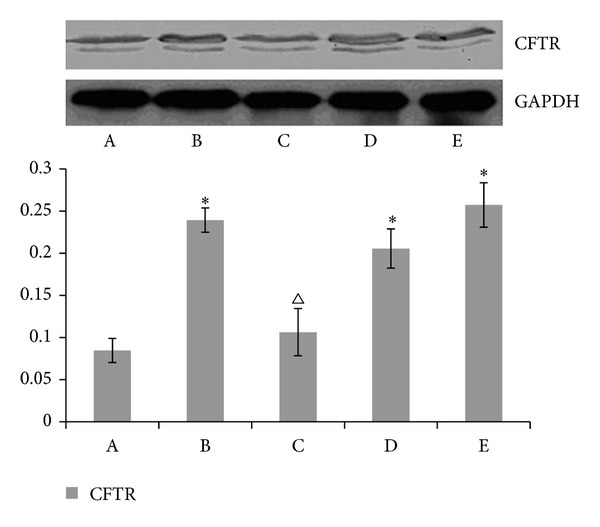
The result of CFTR expression in each rat group. The CFTR protein levels were detected by Western Blot. The CFTR expression levels are expressed as a ratio to control levels. The results are representative of three separate determinations. Compared with the blank group, *P < 0.01 with the modeling group ^▵^ *P* < 0.01.

**Figure 3:**
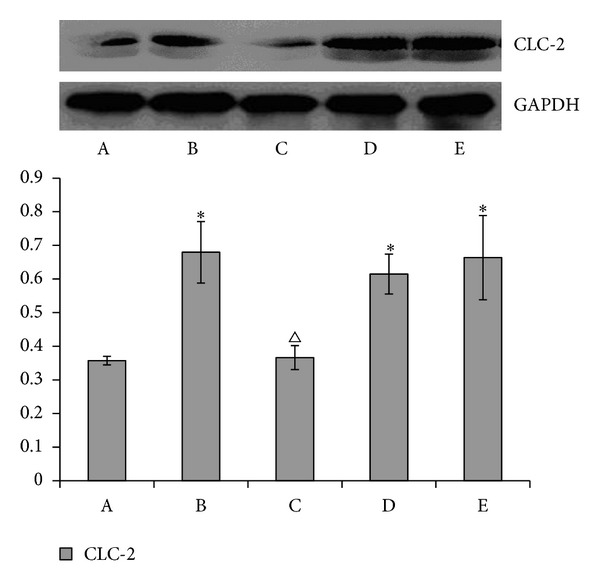
The result of CLC-2 expression in each rat group. The CLC-2 protein levels were detected by Western Blot. The CLC-2 expression levels are expressed as a ratio to control levels. The results are representative of three separate determinations. Compared with the blank group, *P < 0.01 with the modeling group^▵^ *P* < 0.01.

**Table 1:** The amplitude values comparison of T wave in rats ECG before and after modeling.

Group	The amplitude value of T wave (mv)	
	Before	After
MG*	0.16 ± 0.06	−0.08 ± 0.17
NG^#^	0.14 ± 0.06	−0.23 ± 0.18
LG*	0.13 ± 0.04	−0.14 ± 0.16
NA*	0.13 ± 0.62	−0.21 ± 0.71

MG: modeling control group, NG: Neiguan (PC-6) treatment group, LG: Lieque (LU-7) control group, NA: nonacupoint control group. All values are means ± S.E.M. (*n* = 10).*P < 0.05, before and after modeling in the same group. ^#^ *P* < 0.01, before and after modeling in the same group.

**Table 2:** The results of the level of rats serum SOD ($\overset{-}{x}\pm s$).

Group	*n*	SOD (U/mL)
CG	8	66.08 ± 6.90
MG	11	47.30 ± 11.78*
NG	14	61.78 ± 7.56^#^
LG	10	48.20 ± 12.00*
NA	11	48.74 ± 11.52*

CG: blank control group, MG: modeling control group, NG: Neiguan (PC-6) treatment group, LG: Lieque (LU-7) control group, NA: nonacupoint control group. *P < 0.01 versus CG, ^#^ *P* < 0.01 versus MG.

**Table 3:** The results of the contents of rats serum MDA ($\overset{-}{x}\pm s$).

Group	*n*	MDA (nmol/mL)
CG	8	0.15 ± 0.01
MG	11	0.19 ± 0.01*
NG	14	0.13 ± 0.02^#^
LG	10	0.19 ± 0.01*
NA	11	0.17 ± 0.04*

CG: blank control group, MG: modeling control group, NG: Neiguan (PC-6) treatment group, LG: Lieque (LU-7) control group, NA: nonacupoint control group. *P < 0.05 versus CG, ^#^ *P* < 0.05 versus MG.
